# Implications of cellular prion protein in tumor biology

**DOI:** 10.3389/fonc.2026.1906977

**Published:** 2026-09-28

**Authors:** Ahmed Damra

**Affiliations:** Department of Pathology, Case Western Reserve University, Cleveland, OH, United States

**Keywords:** colorectal cancer, cellular prion protein, pro-PrP, filamin A, apoptosis resistance, therapy resistance, epithelial-mesenchymal transition

## Abstract

Prion proteins (PrP) are primarily known for their role in neurodegenerative conditions like Creutzfeldt-Jakob Disease, where the misfolded, pathogenic isoform PrPSc propagates by using the normal cellular prion protein, PrP^c^, as a template, leading to aggregation and neuronal death. PrP^c^ is a widely expressed glycoprotein that, beyond its role in prion disease, has emerged in specific contexts as a modulator of several hallmark cancer processes, including proliferation, apoptosis resistance, cellular signaling, adhesion, survival, and motility. This paper explores current evidence on how PrP^c^, and its misprocessed isoform pro-PrP, contribute to tumor progression across multiple cancer types, using colorectal cancer (CRC) as a unifying disease model to integrate mechanisms that are otherwise dispersed across the literature. This review will examine PrP^c^’s role in sustaining proliferative signaling, resisting apoptosis, promoting migration and metastasis through its interaction with filamin A (FLNa), and contributing to therapy resistance. The goal is to consider the extent to which these mechanisms converge specifically in CRC, and what this means for PrP^c^’s potential as a diagnostic and therapeutic target.

## Introduction

Prion diseases, such as Creutzfeldt-Jakob Disease (CJD), are a group of fatal neurodegenerative disorders caused by the misfolding of a normally benign protein. The pathogenic isoform, PrPSc, propagates by using the normal cellular prion protein, PrP^c^, as a template, leading to aggregation and neuronal death. PrP^c^ is a widely expressed glycoprotein that is normally localized to the outer leaflet of the plasma membrane by a highly conserved glycosylphosphatidylinositol (GPI) anchor. While PrP^c^’s role in neurodegeneration is well established, a growing body of literature has implicated this same protein in cancer biology, where it appears to support tumor cell proliferation, survival, migration, and resistance to therapy (**Figure 1**). This review synthesizes that literature, using colorectal cancer as a model to examine these mechanisms.

**Figure 1:**
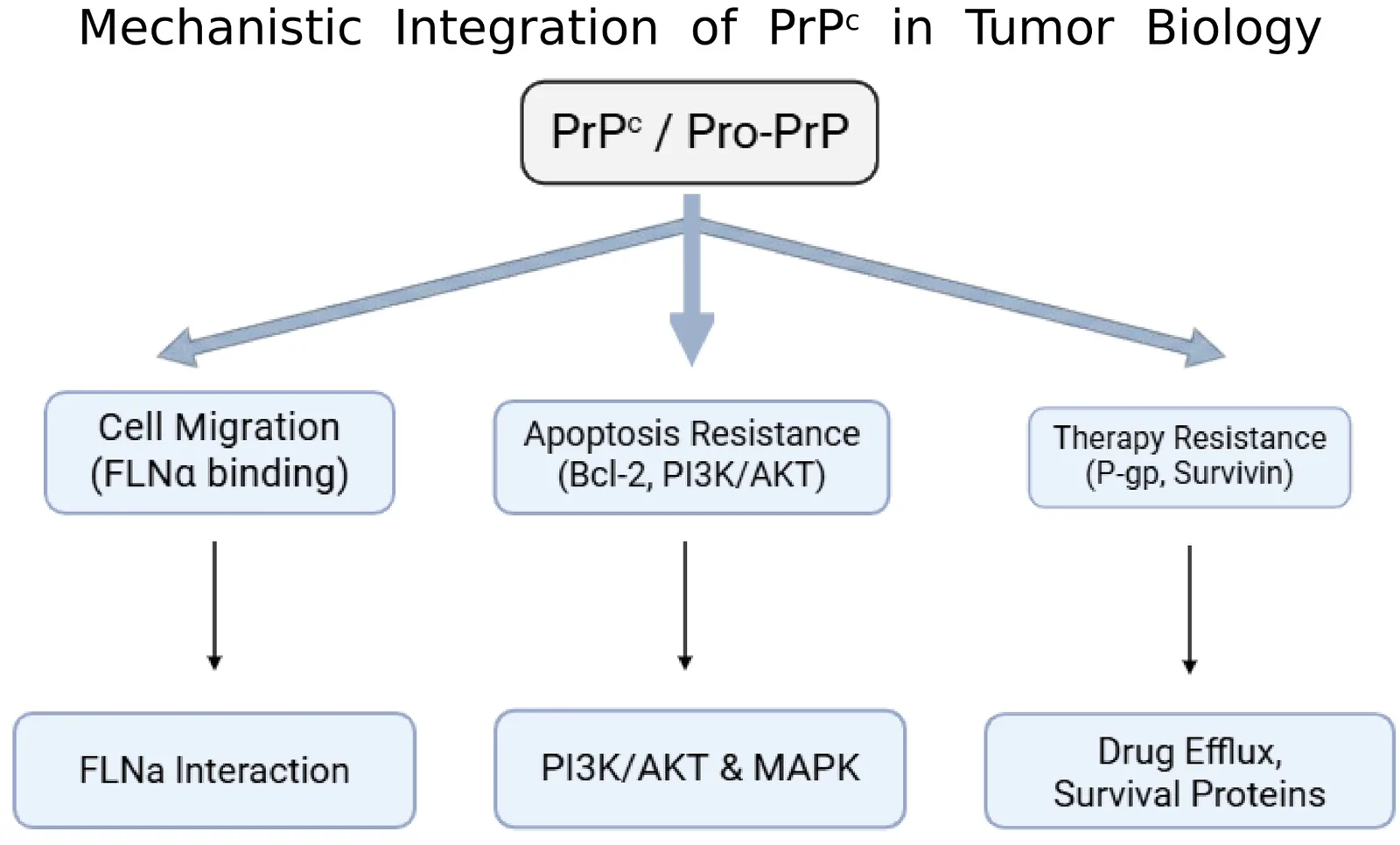
Mechanistic integration of PrP^c^ in tumor biology. PrP^c^ and its misprocessed isoform, pro-PrP, converge on three mechanisms of tumor progression examined throughout this review: binding to filamin A (FLNa) drives cytoskeletal remodeling and cell migration; modulation of Bcl-2 and PI3K/AKT signaling confers resistance to apoptosis; and upregulation of P-glycoprotein (P-gp) and survivin contributes to therapy resistance. Each of these mechanistic axes is examined in detail in the sections that follow.

Research has also identified a contribution to cancer cell migration and invasion by investigating PrP^c^ interaction with filamin A (FLNa), an actin-binding cytoskeletal scaffold protein. Under normal conditions, PrP^c^ is properly processed and GPI-anchored, and this mature form does not interfere with FLNa’s role in organizing the cytoskeleton for stable adhesion. However, in some cancer cells, this process is disrupted, resulting in the expression of an immature form known as pro-PrP. This form retains the GPI-anchor signal sequence (GPI-PSS) that is normally cleaved during maturation, and it is this retained sequence that allows pro-PrP to bind FLNa directly, disrupting its normal cytoskeletal organization and promoting a more migratory, invasive cell state. Its overexpression in cancers across the spectrum, including glioblastoma, osteosarcoma, and prostate cancer, is correlated with a worse prognosis (1), highlighting the potential as both a diagnostic biomarker and a druggable target. Small molecules such as quinacrine and chlorpromazine were originally developed to block PrP^c^→PrPSc conversion in prion disease models; however, both failed to improve survival in human prion-disease clinical trials. Antibody- and RNAi-based strategies that directly target PrP^c^ expression or its specific protein interactions (e.g., with FLNa or P-glycoprotein) have shown promise, and represent a more mechanistically grounded starting point for PrP^c^-directed oncology therapeutics (2–4).

Knockdown of PrP^c^ has been shown to sensitize resistant cells to TRAIL- and chemotherapy-induced apoptosis, suggesting a role for PrP^c^ in treatment response (5). Despite these advances, many of the mechanisms still remain unclear due to ambiguity surrounding PrP^c^’s functions, particularly the distinction between mature PrP^c^ and pro-PrP, which is often blurred in the literature even though the two isoforms appear to contribute to cancer behavior through different mechanisms.

This review draws on peer-reviewed literature identified through PubMed and Google Scholar searches (terms including “PrP^c^,” “pro-PrP,” “prion protein AND cancer,” “filamin A AND cancer,” and “PrP^c^ AND colorectal cancer”), supplemented by manual screening of reference lists from key primary studies and from prior reviews on this topic (3, 4, 6). Rather than surveying PrP^c^’s oncogenic associations broadly across cancer types, as these existing reviews already do well, this review narrows its focus in three ways: it separates the mechanistic contributions of mature, GPI-anchored PrP^c^ from those of the misprocessed pro-PrP isoform throughout, rather than treating the two interchangeably; it traces proliferation, apoptosis resistance, migration, and therapy resistance — largely characterized in individual cell-line and animal models — as they converge within a single disease model, using colorectal cancer to make those connections concrete; and it weighs disconfirming and context-dependent evidence alongside the pro-tumorigenic findings, rather than presenting PrP^c^’s role as uniform across cancer types and stages.

## PrP^c^ and sustained proliferation

PrP^c^ has been reported to enhance pathways such as PI3K/Akt and MAPK/ERK, and other raft-associated signaling pathways, across several tumor cell-line models (7). In gastric cancer cells, elevated PrP^c^ expression is associated with increased phosphorylation of Akt and downstream activation of pathways that support cell cycle progression and resistance to apoptosis; inhibition of PI3K/Akt reverses these effects, supporting the interpretation that PrP^c^ functions upstream of this proliferative pathway (4, 8). Similar associations between PrP^c^ expression and enhanced proliferative signaling have been reported in glioblastoma and breast cancer cell lines, where PrP^c^ knockdown reduces proliferation rates and sensitizes cells to growth arrest, further supporting its role as a proliferative driver and one that is associated with worse prognosis (4).

Beyond direct pathway activation, PrP^c^ has also been implicated in supporting the metabolic and survival demands of rapidly dividing cancer cells, including resistance to endoplasmic reticulum (ER) stress that would otherwise trigger growth arrest or apoptosis in response to the metabolic strain of uncontrolled proliferation. By buffering this stress, PrP^c^ may allow tumor cells to sustain high proliferation rates that would otherwise be self-limiting, further enhancing the ability of cancer cells to survive therapeutic stress (9).

Taken together, these findings position PrP^c^ not merely as a passive marker of proliferative cancer cells, but as an active contributor to the signaling architecture that sustains uncontrolled growth. This is consistent with the broader role of PrP^c^ as an upstream regulator of canonical growth pathways.

## PrP^c^ and resistance to cell death (apoptosis)

One of the most well-characterized roles of PrP^c^ in cancer biology is its contribution to apoptosis resistance. In the cell-line models studied to date, PrP^c^ overexpression has been associated with upregulation of anti-apoptotic proteins (such as Bcl-2, survivin, and IAPs) and downregulation of pro-apoptotic factors (such as Bax), a shift that would be expected to limit activation of the caspase cascade. Additionally, the mechanisms by which PrP^c^ can activate PI3K/Akt and MAPK/ERK pathways contribute to survival signaling and may also inhibit apoptotic responses (4, 7).

This anti-apoptotic function was first characterized outside the cancer context entirely: PrP^C^ was shown to prevent Bax-mediated apoptosis in human primary neurons, protecting cells from Bax-induced death (10). This early finding established PrP^c^ as more than a passive bystander in cell death pathways, and set the stage for later work examining whether this same protective function is co-opted by cancer cells, where it functions as an endogenous inhibitor of programmed cell death.

In breast and gastric cancer models, silencing PrP^c^ sensitizes cells to apoptosis induced by chemotherapeutic agents, indicating that its normal function includes dampening stress-induced cell death pathways that would otherwise be triggered by treatment. This effect is not limited to a single drug class; PrP^c^ knockdown enhances sensitivity to TRAIL-, doxorubicin-, and paclitaxel-induced apoptosis across multiple cell lines (11), suggesting a broadly protective role that cancer cells exploit rather than a mechanism specific to any one therapeutic agent, actively contributing to cancer cell survival under treatment stress.

Mechanistically, PrP^c^ appears to interfere with the intrinsic apoptotic pathway at multiple points, from stabilizing mitochondrial membrane integrity to modulating downstream caspase activation, allowing cells to bypass apoptosis in the presence of treatment.

Across neuronal and cancer models, PrP^c^ adjusts the balance of Bcl-2 family proteins (upregulating anti-apoptotic members such as Bcl-2, survivin, and IAPs while downregulating pro-apoptotic factors such as Bax), interferes with Bax-driven activation of the caspase cascade, and maintains pro-survival signaling through PI3K/Akt and MAPK/ERK, contributing directly to therapeutic resistance (4).

## PrP^c^ and migration/metastasis

PrP^c^’s contribution to cancer progression extends beyond survival signaling into the physical processes that allow tumor cells to invade surrounding tissue and metastasize. Central to this role is PrP^c^’s interaction, in its misprocessed pro-PrP form, with filamin A (FLNa), a cytoskeletal scaffold protein that organizes actin filaments and links them to membrane receptors such as integrins. This interaction reorganizes the cytoskeleton in a way that favors motility over stable adhesion, a shift that appears to be required for directed cell migration in tumorigenic cells.

As seen in **Figure 2**, under normal conditions (left), properly processed PrP^c^ is properly anchored to the cell membrane through the use of a GPI anchor and is unable to interfere with cytoskeletal organization directly. Filamin A (FLNa) links β1 integrin to the actin cytoskeleton, supporting regulated cell adhesion and controlled migration. Conversely (right), misprocessed pro-PrP retains the GPI-PSS segment, allowing it to anchor to the membrane, but it does so abnormally. This abnormal attachment allows pro-PrP to interact abnormally with cytoskeletal regulatory proteins, particularly Filamin A (FLNa), which normally links integrins to the actin cytoskeleton and helps stabilize cell structure (12). In this context, pro-PrP binding alters how FLNa organizes actin filaments and connects to integrins. Research into this aberrant binding further elucidates the role of pro-PrP, specifically, in this process (13). Instead of maintaining stable adhesion, the interactions cause the cytoskeleton to be more dynamic, allowing cells to detach and reattach as needed for cell migration readily. In melanoma models, pro-PrP was shown to bind directly to FLNa through its retained GPI-PSS segment, and this interaction increased association between FLNa and β1 integrin, forming a complex that enhanced cell movement. When pro-PrP expression was reduced in FLNa-positive cells, FLNa became mislocalized and actin organization was disrupted, leading to impaired cell spreading and migration. Conversely, the presence of pro-PrP promoted cytoskeletal rearrangement and increased motility. This effect could be blocked using a peptide that mimics the pro-PrP GPI-PSS region, supporting the conclusion that the pro-PrP–FLNa interaction is necessary for this behavior. Together, these findings suggest that misprocessed pro-PrP does not just associate with metastatic behavior but contributes to it by shifting the cytoskeleton from a stable, adhesive state to one that supports continuous remodeling, migration, and invasion (13, 14).

**Figure 2:**
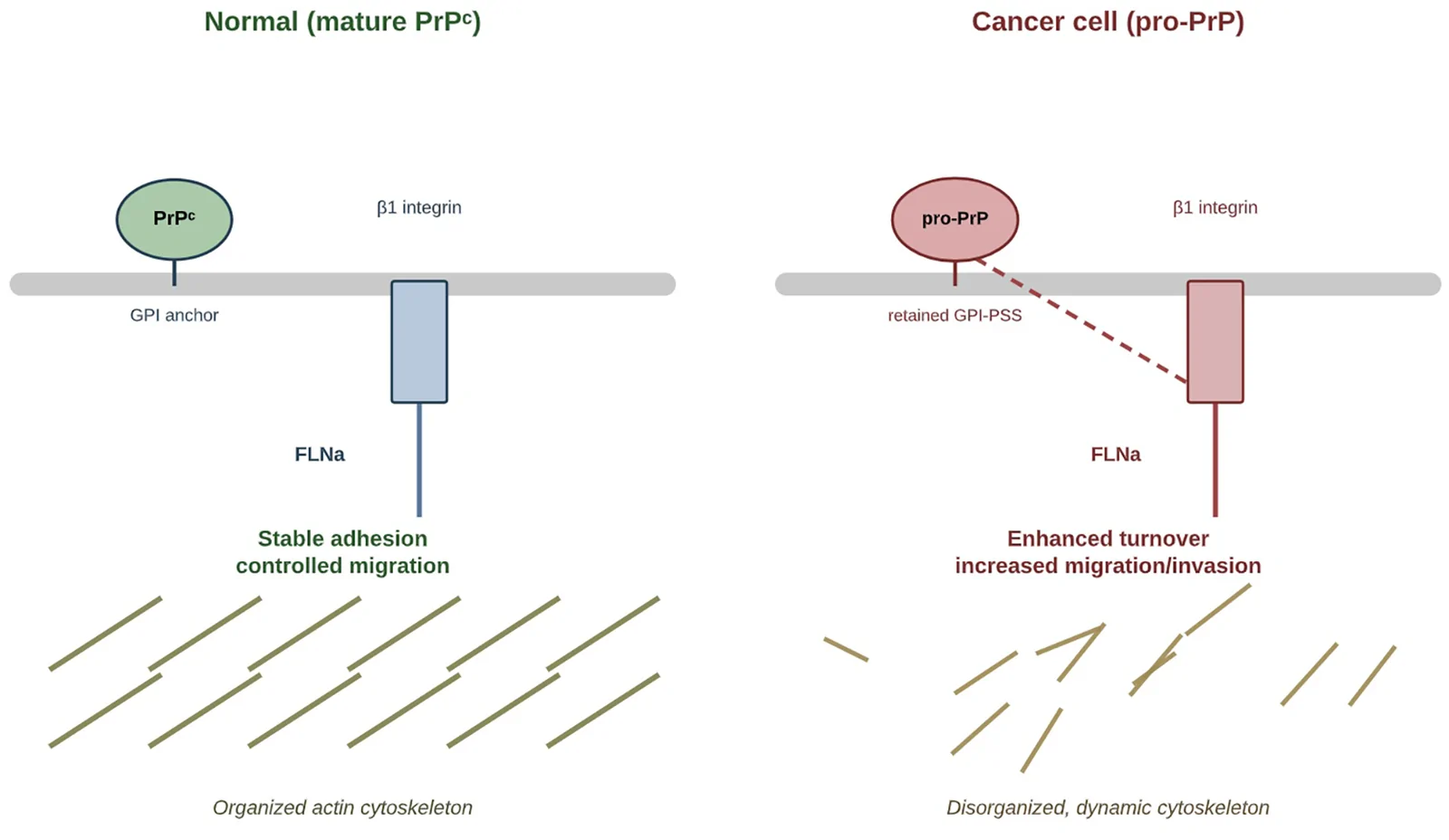
Effect of pro-PrP on filamin A–dependent cytoskeletal organization. Left: In cells expressing properly processed, GPI-anchored PrP^c^, FLNa links β1 integrin to the actin cytoskeleton in a stable configuration that supports regulated adhesion and controlled migration (13, 15). Right: In cells expressing pro-PrP, the retained GPI-PSS motif enables aberrant binding to filamin A (FLNa), disrupting its normal scaffolding function and producing a disorganized, dynamically remodeling cytoskeleton that favors enhanced turnover, migration, and invasion.

Another study examining pancreatic BxPC-3 cancer cells showed that a deficiency in GPI-anchor attachment leads to accumulation of pro-PrP, which in turn increases cancer cell motility. The data indicate that a loss of proper GPI anchoring correlates with better migration and the pro-PrP–FLNa mechanism in this phenotype. This further supports the hypothesis that pro-PrP binding to FLNa contributes to the heightened motility, consistent with earlier findings (15, 16).

The effect of pro-PrP is not limited to physically reorganizing the cytoskeleton. The interaction between pro-PrP and FLNa has also been found to alter how integrins function as signaling molecules, shifting the cell from stable adhesion to continuous turnover. Instead of maintaining a secure attachment to the extracellular matrix, pro-PrP causes adhesion to be short-lived and unsustained, which is ideal for metastatic movement. This matters because FLNa functions as a scaffold for multiple pathways, including those associated with integrins and small GTPases. This means that when pro-PrP disrupts its normal organization, the consequences extend beyond cytoskeletal mechanics into broader pro-migratory signaling (12).

In breast cancer cell lines, FLNa-deficient cells show impaired invasion despite retaining pro-PrP expression, indicating that FLNa itself is required as a downstream effector rather than merely a passive binding partner; without functional FLNa, pro-PrP’s pro-migratory effects cannot be realized. Restoring FLNa expression in these cells re-establishes pro-PrP’s ability to promote invasion, and in FLNa-positive cells, pharmacological inhibition of filamin can directly reduce metastatic potential regardless of pro-PrP status (17), suggesting that FLNa sits at a convergence point where it can directly amplify the signaling environment.

In breast cancer models, PrP^c^ has also been shown to regulate invasion and migration through activation of matrix metalloproteinase-9 (MMP-9), an enzyme that degrades extracellular matrix components and facilitates tissue invasion. PrP^c^ knockdown reduces MMP-9 activity and correspondingly reduces invasive capacity, while PrP^c^ overexpression increases MMP-9 expression via ERK1/2 signaling, allowing cells to degrade surrounding matrix better to support migration (18).

Epithelial-to-mesenchymal transition (EMT) represents another route through which PrP^c^ contributes to metastatic behavior. EMT is a process by which epithelial cells lose polarity and cell-cell adhesion, taking on mesenchymal characteristics that favor motility and invasiveness; cancer cells frequently reactivate this normally developmental program in order to readily detach and migrate (19).

PrP^c^ contributes to this transition by affecting the gene expression behind EMT, and pathogenically impacting cytoskeletal behavior that defines EMT. General EMT programs are characterized by suppression of E-cadherin alongside induction of mesenchymal markers such as N-cadherin and the transcription factor TWIST (20). Given PrP^c^’s engagement of the PI3K/AKT, MAPK/ERK, and Wnt/β-catenin pathways discussed throughout this review, studies show a convergence in CRC specifically, though a direct, PrP^c^-specific report of E-cadherin/N-cadherin/TWIST regulation in colorectal cancer must be investigated further. This change in gene expression would be reinforced by the same PI3K/AKT and MAPK/ERK signaling described above, which helps stabilize these transcriptional programs and support continued survival during the transition. At the structural level, these changes are supported by PrP^c^’s effects on the cytoskeleton. Through interactions with FLNa, pro-PrP alters actin organization and promotes cytoskeletal remodeling that favors movement rather than stable adhesion (13); separately, PrP^c^-driven Akt signaling contributes to survival during this transition, as discussed above.

In one study investigating melanoma, pro-PrP (GPI-absent precursor) is not found within normal melanocytes but can be detected within tumor cells. Additionally, as pro-PrP levels increase in the cell, tumors become increasingly more invasive (13), suggesting that acquisition of the pro-PrP phenotype itself contributes to the transition from a localized, contained tumor to one capable of metastasis. A similar relationship has been observed in pancreatic cancer models, where higher pro-PrP levels correspond to increased expression of mesenchymal markers and reduced expression of epithelial markers, reinforcing the shift toward an invasive, metastatic phenotype (21).

Taken as a whole, the evidence discussed in this section positions pro-PrP, acting through its interaction with FLNa, as a central driver of the cytoskeletal remodeling required for cancer cell migration, while mature PrP^c^ contributes a complementary layer of transcriptional and signaling support. Together, these mechanisms allow tumor cells to detach, move, and reattach in order to spread successfully.

## PrP^c^ and therapy resistance

PrP^c^ contributes to therapy resistance through at least two distinct but overlapping mechanisms: direct interference with drug efflux and transport, and suppression of the apoptotic pathways that chemotherapeutic agents rely on to kill cancer cells. Both mechanisms allow tumor cells to survive treatment that would otherwise be effective, contributing to both mechanisms of resistance.

PrP^c^’s role in reducing a drug’s intracellular concentration can be understood through its interaction with drug efflux machinery. In multidrug-resistant breast cancer cells treated with paclitaxel, PrP^c^ physically interacts with P-glycoprotein (P-gp), a transmembrane efflux pump, and this interaction enhances P-gp’s ability to export the drug out of the cell before it can exert cytotoxic effects. PrP^c^-expressing cells can limit drug accumulation; however, their resistance mechanism is twofold: PrP^c^ appears to help organize a localized environment that favors survival under chemotoxic stress (22).

Beyond efflux, PrP^c^ also suppresses the apoptotic response that would normally be triggered once a drug reaches its intracellular target. In cells resistant to adriamycin and TRAIL, PrP^c^ silencing restores sensitivity to these agents, indicating that its presence actively blocks the downstream apoptotic signaling these drugs depend on, rather than drug-specific pathways.

In colorectal cancer specifically, elevated PrP^c^ expression is associated with sustained activation of the PI3K/Akt pathway in cells exposed to 5-fluorouracil (5-FU), a first-line chemotherapeutic agent for CRC. This continued survival signaling in the presence of treatment suggests that PrP^c^ helps cells tolerate the metabolic and genotoxic stress induced by 5-FU exposure, and that ongoing PI3K/Akt activity is required to maintain the signaling environment that allows resistance to persist (23).

Taken together, these findings position PrP^c^ as a multi-pronged contributor to therapy resistance, one that operates by limiting intracellular drug concentration on one hand, and by suppressing the apoptotic machinery that treatment is meant to trigger on the other. This dual role likely contributes to the difficulty of achieving durable responses in PrP^c^-high tumors, and underscores the protein’s relevance to treatment failure and disease progression (3).

## Colorectal cancer as a model of PrP^c^-driven tumor behavior

Collectively, the preceding sections position PrP^c^ as a membrane-associated signaling modulator capable of enhancing PI3K/AKT and MAPK/ERK activation, promoting resistance to apoptosis, and facilitating cytoskeletal remodeling and migration. Colorectal cancer (CRC) provides a useful model in which to examine how these mechanisms converge in a single disease context, given the extent to which CRC progression is shaped by many of the same signaling pathways, and its cells are especially responsive to PrP^c^-associated stromal cues that modulate responses to stromal/CAF-derived cues.

CRC progression is strongly shaped by dysregulated signaling pathways such as Wnt/β-catenin, EGFR/RAS/RAF/MEK/ERK, and PI3K/AKT/mTOR, many of which overlap directly with the pathways PrP^c^ has already been shown to engage in other cancer contexts discussed above (24). The link between PrP^C^ and the mesenchymal CRC subtype was first established by Le Corre et al. Using two large patient cohorts (n = 566 and n = 1682), they found that PRNP expression is selectively elevated in the C4/CMS4 subgroup and is associated with worse overall and relapse-free survival. Mechanistically, PrP^c^ drove this phenotype through two parallel routes in C4/CMS4 colon cancer cells: silencing PrP^c^ reduced TAZ levels and shifted the balance toward inactive, phosphorylated YAP, lowering expression of several YAP/TAZ target genes central to the C4/CMS4 signature; separately, PrP^c^ silencing also cut TGFB1 transcription and secreted TGFβ1, and adding back recombinant TGFβ1 rescued C4/CMS4 gene expression in the PrP^c^-depleted cells. Overexpressing PrP^c^ in a non-C4/CMS4 line produced the opposite effect, increasing both TGFβ1 secretion and C4/CMS4 gene expression. The same group also measured circulating PrP^c^ in plasma from 325 patients with metastatic CRC and found levels nearly double those of healthy controls, with high plasma PrP^c^ independently associated with worse disease control (25). This gives PrP^c^-driven Hippo (YAP/TAZ) and TGFβ signaling as a second, CRC-subtype-specific route into the mesenchymal phenotype, distinct from the PI3K/AKT- and MAPK/ERK-centered mechanisms covered earlier — and sets up the PRNP-CTNNB1-NR3C1 findings below. Building on this, a later study by the same group shows that Wnt-beta-catenin cooperates directly with PrPC and the glucocorticoid receptor (NR3C1) to drive the mesenchymal CMS4 subtype, creating a PRNP-CTNNB1-NR3C1 signature that is overrepresented in the mesenchymal subtype and associated with a shorter time to recurrence in stage III patients (26). PrP^c^’s role in CRC isn’t limited to adhesion and transcriptional reprogramming — it also extends into how these cells handle glucose metabolism. In DLD-1 and SW480 cells, knocking down PrP^c^ caused a drop in glucose transporter 1 (Glut1) expression, and tracing this back showed it runs through Fyn: PrP^c^ knockdown shut off constitutive Fyn kinase activity, which in turn stopped HIF-2α from binding the Glut1 promoter and shutting down Glut1 transcription. Since Glut1 is what feeds the elevated glycolysis (the Warburg effect) CRC cells depend on, PrP^c^-depleted cells showed lower glycolytic rate, glucose uptake, and viability, and PrP^c^ knockdown slowed tumor growth in a xenograft model as well (27). In patient tissue, PrP^c^ expression tracked with histological grade across 130 primary colorectal adenocarcinomas and correlated tightly with Glut1 expression (Pearson’s R = 0.91), tying this metabolic mechanism directly to disease severity. This Fyn-HIF-2α-Glut1 axis adds a metabolic angle to the proliferation and survival mechanisms already covered above, suggesting that PrP^c^ may help fuel CRC cells to survive and adhere.

EMT is a recurring theme in CRC progression as well, and in CRC, it is closely tied to Wnt/β-catenin signaling as well as cross-talk with MAPK and PI3K pathways (28). The same FLNa-dependent cytoskeletal remodeling discussed in the Migration/Metastasis section applies directly to CRC cells (29), where pro-PrP accumulation has been associated with increased invasive capacity through the basement membrane, a critical early step in CRC metastasis to the liver (30–32). Loss of E-cadherin and gain of mesenchymal markers, driven partly by Wnt/β-catenin activity, allow EMT-like behavior to persist once it begins.

CRC cells also display considerable plasticity, moving between epithelial and mesenchymal states depending on microenvironmental cues, and PrP^c^ may act as a stabilizer of that adaptability by reinforcing survival pathways such as PI3K/AKT and ERK, allowing cells to shift readily between growth, survival, and movement (33).

The tumor microenvironment (TME) further shapes how these mechanisms play out in CRC. Cancer-associated fibroblasts (CAFs) secrete factors that can modulate Wnt signaling and influence whether tumor cells favor an EMT-like state (34), and PrP^c^-expressing tumor cells may be particularly responsive to these stromal cues given its role in amplifying upstream signaling inputs. This interplay between tumor cells and their surrounding stroma helps CRC cells preserve their malignant phenotype under pressure (35).

CD44-positive cancer stem cells expressing PrP^c^ represent a particularly aggressive subpopulation within CRC tumors, one that combines stemness with the metastatic machinery discussed throughout this review, allowing these cells to sustain invasion and survival under therapeutic stress (36).

As described in the Therapy Resistance section above, PrP^c^ helps maintain PI3K/AKT signaling while suppressing stress-induced apoptosis in 5-FU–exposed CRC cells, making its role in CRC-specific resistance especially important. 5-FU treatment itself has been shown to promote a WNT3-driven stemness phenotype in CRC cells, independent of PrP^c^, but the overlap between this WNT3 pathway and PrP^c^’s own engagement of Wnt/β-catenin signaling suggests the two may act cooperatively in cells that survive treatment (37). As shown in **Figure 3**, PrP^c^ sits at the convergence of extracellular signaling inputs and amplifies signaling networks that collectively support tumor progression (38).

**Figure 3:**
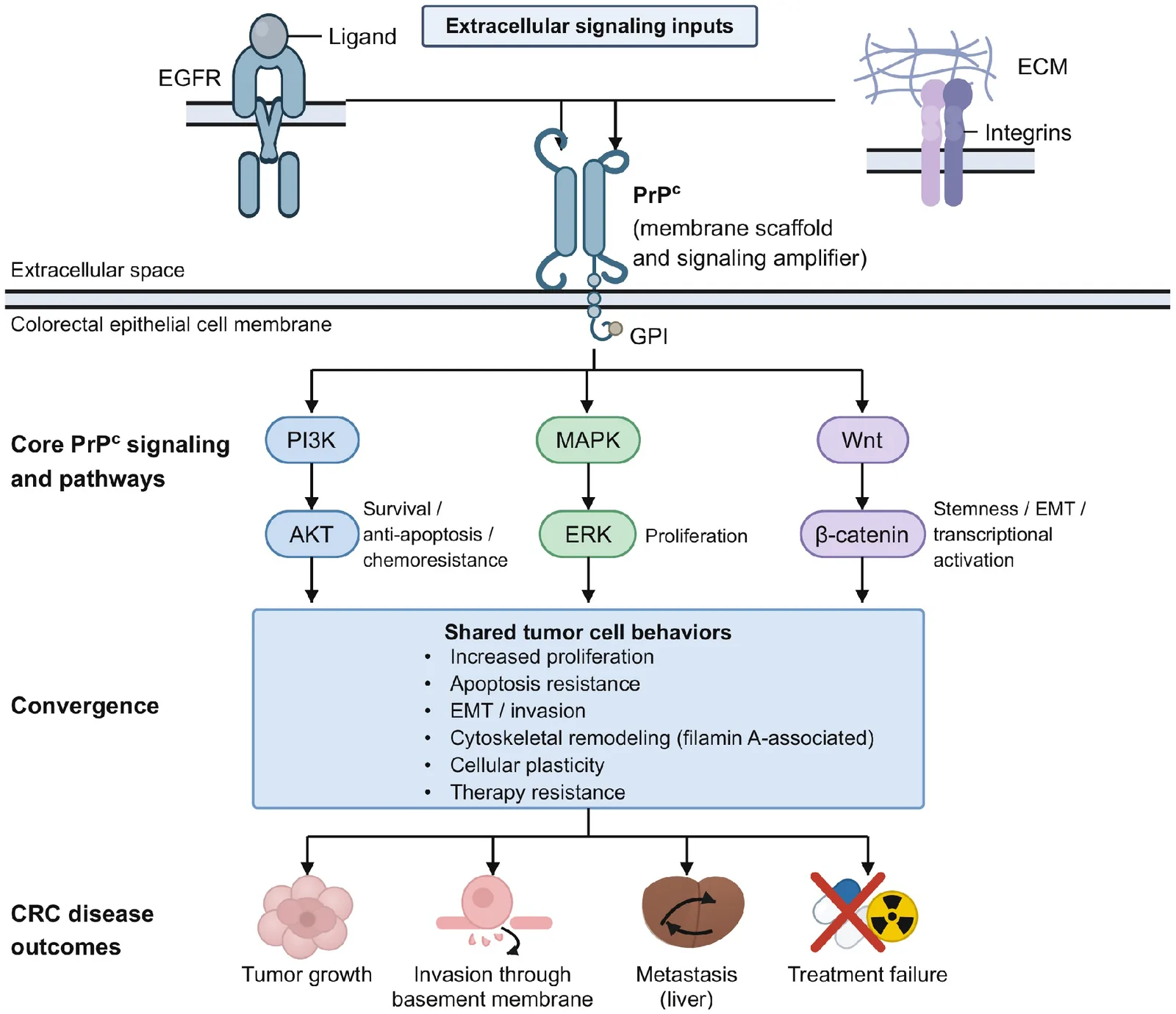
Convergence of PrP^c^-associated signaling on colorectal cancer disease behaviors. Extracellular inputs, including EGFR ligand engagement and integrin–extracellular matrix (ECM) contact, converge on membrane-associated PrP^c^, which functions as a signaling scaffold and amplifier. PrP^c^ engagement of the PI3K/AKT, MAPK/ERK, and Wnt/β-catenin pathways supports survival and chemoresistance, proliferation, and stemness/EMT-associated transcriptional programs, respectively. These converge on a shared set of tumor cell behaviors (proliferation, apoptosis resistance, EMT/invasion, filamin-A–associated cytoskeletal remodeling, cellular plasticity, and therapy resistance) that collectively drive CRC-specific outcomes, including tumor growth, invasion through the basement membrane, hepatic metastasis, and treatment failure.

Taken together, the convergence of PrP^c^-associated mechanisms in CRC — proliferative signaling, apoptosis resistance, cytoskeletal remodeling, and therapy resistance — illustrates why this cancer type serves as a useful model for understanding PrP^c^’s broader role in tumor biology. Rather than acting through a single isolated pathway, PrP^c^ appears to reinforce multiple, mutually supporting mechanisms simultaneously, which may explain the difficulty of targeting it therapeutically without addressing the constraints imposed by the surrounding microenvironment.

## Context-dependent evidence

The evidence discussed above is drawn from studies that detect a pro-tumorigenic role for PrP^c^, and it should not be read as establishing that PrP^c^ acts uniformly in this direction across cancer types, expression levels, or disease stages. Several considerations argue for a more cautious, context-dependent interpretation.

First, the prognostic association between PrP^c^ expression and clinical outcome is not consistent across all reported cohorts. A 2024 re-evaluation of PrP^c^ expression in primary esophageal squamous cell carcinoma and primary hepatocellular carcinoma reported a more complex relationship with patient prognosis than earlier single-cohort studies had suggested (39), indicating that the direction and strength of the PrP^c^–prognosis association cannot be assumed to generalize from one cancer type to another.

Furthermore, the core biochemical functions attributed to PrP^c^ throughout this review — modulation of the Bcl-2/Bax balance, engagement of PI3K/AKT and MAPK/ERK signaling, and cytoskeletal scaffolding via filamin A — are physiological, context-dependent survival and homeostatic mechanisms rather than dedicated oncogenic circuitry. The same anti-apoptotic function that protects a malignant cell from treatment-induced death is, in a normal or pre-malignant cell, indistinguishable from ordinary cytoprotection; indeed, the original characterization of PrP^c^’s anti-apoptotic activity was made in primary neurons, not cancer cells (10). Whether and at what stage this same activity becomes selectively advantageous to a transformed cell, as opposed to simply preserving normal tissue, is not resolved by the existing literature and represents a meaningful gap rather than an assumption this review can settle.

Finally, the signaling pathways PrP^c^ is reported to amplify are themselves not unidirectional. PI3K/AKT, MAPK/ERK, and Wnt/β-catenin signaling can each promote either proliferation and survival or, depending on cellular context, differentiation and growth arrest. A protein that amplifies these pathways would be expected to inherit this same context-dependence rather than uniformly favor malignant behavior, particularly outside the specific cell-line systems in which each finding was generated.

None of this undermines the mechanistic findings summarized above. It does argue against treating PrP^c^ as a uniformly pro-tumorigenic factor whose therapeutic targeting would be expected to behave identically across cancer types, expression contexts, and stages of disease — a point this review returns to in the Conclusion.

## Therapeutic and diagnostic potential

Given its involvement across so many hallmarks of cancer progression, PrP^c^ has attracted interest as both a prognostic biomarker and a potential therapeutic target, particularly in CRC where its mechanistic contributions have been most thoroughly mapped in this review.

Clinically, high PrP^c^ expression correlates with advanced stage, distant metastasis (liver or lung), and markedly poorer survival (median 12 months versus more than 5 years in low-expressors) — findings drawn from a tissue microarray analysis of 309 colorectal tumor specimens (36). Targeting PrP^c^ with monoclonal antibodies has been shown to reduce metastasis in orthotopic models, further supporting its potential as a therapeutic target in metastatic CRC (36). Diagnostically, tumor and even circulating PrP^c^ levels may offer meaningful insight into how a tumor behaves under therapeutic pressure.

Looking ahead, therapeutic strategies that combine PrP^c^-targeted approaches with existing standard-of-care treatments may offer the greatest promise, particularly given PrP^c^’s role in multiple, overlapping resistance mechanisms. Rather than positioning PrP^c^-targeted therapy as a replacement for existing regimens, the evidence discussed throughout this review suggests it may be most useful as a sensitizing adjunct, one that addresses the specific ways tumor cells survive, proliferate, and spread (3).

## Conclusion

This review has examined the evidence linking PrP^c^, and its misprocessed isoform pro-PrP, to several hallmarks of cancer progression: sustained proliferative signaling, resistance to apoptosis, migration and metastasis via the FLNa interaction, and multi-pronged therapy resistance. Using colorectal cancer as a unifying model, these mechanisms converge to support a malignant phenotype that is difficult to treat using conventional approaches alone.

It is important to emphasize, however, that much of this evidence remains preclinical and is drawn predominantly from *in vitro* cell-line models, with more limited support from *in vivo* and clinical data. PrP^c^ is more accurately characterized as a signaling modulator that amplifies existing oncogenic pathways, rather than as an oncogenic initiator in its own right; its contribution appears to be one of reinforcement and context-dependent amplification rather than causation. This is consistent with the context-dependent and, in some settings, disconfirming evidence discussed above, which argues against treating PrP^c^ as a uniformly pro-tumorigenic factor.

PrP^c^’s therapeutic and diagnostic value, while conceptually strong, remains experimentally preliminary. Future work should prioritize *in vivo* validation of the mechanisms described here, particularly in CRC, along with better-powered clinical cohorts that can establish reliable, directly citable prognostic associations, and continued efforts to distinguish the specific contributions of mature PrP^c^ from those of pro-PrP across cancer types.
